# Common mental disorder in Nyanza province, Kenya in 2013 and its associated risk factors –an assessment of change since 2004, using a repeat household survey in a demographic surveillance site

**DOI:** 10.1186/s12888-015-0693-5

**Published:** 2015-12-09

**Authors:** Rachel Jenkins, Caleb Othieno, Linnet Ongeri, Peter Sifuna, Michael Ongecha, James Kingora, David Kiima, Raymond Omollo, Bernhards Ogutu

**Affiliations:** 1 Health Services and Population Research Department, Institute of Psychiatry, Kings College London, de Crespigny Park, London, SE 5 8AF UK; 2 Department of Psychiatry, University of Nairobi, Nairobi, Kenya; 3 Kenya Medical Research Institute, Kisumu, Kenya; 4 Kenya Medical Training College, Nairobi, Kenya; 5 Ministry of Health, Nairobi, Kenya

**Keywords:** Common mental disorder, Gender, Life events, Assets, Employment, Prevalence, Surveillance site, Ten year trends

## Abstract

**Background:**

Repeat household surveys are useful to assess change in prevalence over time, but there have been no repeat surveys of common mental disorder (CMD) in Kenya, or indeed sub-Saharan Africa. Therefore a repeat household survey of CMD and its associated risk factors was conducted in Maseno area, Kisumu county in Kenya, using a demographic surveillance site as the sample frame, in order to test the hypotheses that (a) the prevalence of CMD would increase between 2004 and 2013 due to the intervening political, social and economic pressures; (b) as in 2004, there would be no gender difference in prevalence of CMD.

**Methods:**

One thousand one hundred ninety households were selected, and 1158 adult participants consented to be interviewed with a structured epidemiological assessment while 32 refused to participate in the study interviews, giving a response rate of 97.3 %.

**Results:**

The study found that the overall prevalence of CMD in 2013 was 10.3 %. However, there were significantly higher rates of having any CMD in 2013 if one was female (OR 6.2, *p* < 0.001), divorced/widowed (OR 2.5, *p* < 0.003), aged over 60 (OR 2.3, *p* = 0.052), either self-employed (OR 3.3 *p* < 0.001) or employed (OR 3.3, *p* < 0.001), or belonged to the lowest asset quintile (OR 2.5, *p* = .0.004) after adjusting for other variables significant at the bivariate level. The overall prevalence in 2013 was consistent with that found in 2004, despite intervening political and community turbulence. However, this apparent consistency masks the development of a striking difference in prevalence between the genders. Over the decade 2004–13, the prevalence for men dropped from 10.9 to 3.8 % (*P* = 0.001) and the prevalence for women increased from 10.8 to 17.5 % (*p* = 0.001).

**Conclusion:**

Common mental disorders continue to pose a significant public health burden in Kenya, and gender related vulnerability merits further research and is relevant for health worker training.

## Background

It is helpful for low and middle income as well as rich countries to monitor their population psychiatric morbidity, because of the powerful impact of poor mental health on human, social and economic capital [1]. There is growing international concern about the mental health treatment gap [2] and the need to scale up services to meet mental health needs [3], despite scarce resources [4] aggravated by continued outward migration of psychiatrists [5]. Population based epidemiological surveys are an essential tool for estimating population health, morbidity, co-morbidities, disability, associated risk factors and the extent to which health needs are met by the health services. This information is needed to inform policy and planning [6] for meeting mental health needs both in the general population and in vulnerable groups.

Kenya was a low income country in sub Saharan Africa until 2014 when it became a lower middle income country. Kenya’s Gross Domestic Product (GDP) per capita rose from US$ 399 in 2000, to $520 in 2005 and to US$1040.55 in 2013 [7], although poverty levels remain high at 45.9 % [8]. Kenya’s population is growing rapidly by about 1 million a year. Thus the population increased from 10 million in 1969 to an estimated 38 million in 2004 and 43.2.million in 2013 (extrapolated from the 2009 census) [9]. Nearly 50 % of the population is aged below 15. Life expectancy at birth is 57 years, the adult literacy rate is 87 %, the infant mortality rate is 55 per 1000 live births, the maternal mortality rate is 490 per 100,000 live births and HIV prevalence is currently estimated at 5.6 % [10]. There is rapid urbanisation, the agricultural sector is highly inefficient, and the food supply is vulnerable to catastrophic drought and floods. On the other hand, there is modest but sustained economic growth. GDP Growth rate in Kenya averaged 1.15 % from 2005 until 2014 [11]. There have been recent discoveries of oil and gas.

Nyanza Province, where the study was conducted, is a mainly rural community with strong social networks, spiritual practices and an unprocessed diet, particularly fish from Lake Victoria. However it has relatively high levels of unplanned pregnancies (53 %), and deaths of children under 5 per 1000 live births (14.9 %) [12]. Indeed, Nyanza has the highest rate of teenage pregnancy in Kenya (22.2 %) and high rates of domestic violence (10.3 % of men and 49.5 % of women report domestic violence in Nyanza compared to 8.6 % of men and 38.4 % of women in Kenya as a whole [13]. Nyanza also has considerably higher prevalence, compared to the rest of Kenya, of HIV of 17.7 % in women and 14.1 % in men [14]. All these socioeconomic and health challenges may impact on the mental health of the population. Fishing was a key occupation around Lake Victoria but the invasion of the Lake by water hyacinth has reduced fishing and aggravated poverty and food insecurity. Thus people living in Nyanza are grappling with poverty, the highest rates of HIV, malaria and common childhood diarrhoeal illnesses in the country, and are therefore facing high rates of life threatening conditions, high levels of infant mortality and other bereavements, and high levels of domestic violence, especially the women. Nyanza was the site of significant election violence around the time of the 2007 general election [15]. Furthermore there are local cultural practices that are challenging for women, including widow inheritance and sexual cleansing rituals which increase the risk of exposure to HIV, polygamy, early marriage and early coital debut [16].

Despite the clear need for epidemiological information on population mental health, and a number of household epidemiological studies of mental disorders conducted in Sub-Saharan Africa [17], there has only been one previous epidemiological study of mental health in the general adult population in Kenya reporting on common mental disorder [18] and psychotic symptoms [19]. Therefore the opportunity was taken to conduct this second such survey as part of a wider project to examine the associations between mental disorder, malaria and immunity, the results of which will be reported elsewhere.

The research was conducted as part of an overall collaborative programme of work between the Kenya Ministry of Health and the UK Institute of Psychiatry, Kings College London (KCL) over the last 15 years, (including collaborations with the Kenya Medical Training College (KMTC), the Kenya Medical Research Institute (KEMRI), the Kenya Psychiatric Association, and Great Lakes University) comprising situation appraisal [18–22], studies of traditional healers [23], district health workers [24], community health workers [25], policy development [26], primary care training [27, 28] and its evaluation [29–32]. This repeat epidemiological survey is important to assess the sustained population health needs due to common mental disorder (comprising mixed anxiety depression, depression, anxiety, phobias, and obsessive compulsive disorder) in the general adult population in a region of Kenya which experienced serious election violence in 2007/8 [15].

## Methods

The study design was a community study of the prevalence of common mental disorder (CMD), and its risk factors in the general population in Nyanza Province, near Lake Victoria in Kenya.

### Study population

The sample frame is a subdivision in an endemic area for malaria in Kenya, namely Maseno area in Kisumu County, Nyanza Province in western Kenya (see Fig. 1). Maseno has a population of 70,805 [33]. Females constitute 53 % of the population. The mean household size is 4 people per household with a population density of about 374 people/km^2^. The population is largely young with a mean age of 23 years. The population 0–14 years constitutes 46 %, ages 15–64 years constitute 49 % and ages 65+ years constitute 5 %.

The population is primarily black African, and the languages spoken are Dholuo (which is for the predominant ethnic group Luo), Kiswahili and English. The area is largely rural, with most residents living in villages, which are a loose conglomeration of family compounds near a garden plot and grazing land. The majority of the houses are mud-walled with either grass thatched or corrugated iron-sheet roofs. Water is sourced mainly from community wells (40 %), local streams (43 %) and the lake (5 %) for those mostly living on the shores of Lake Victoria [33]. Most water sources are not chlorinated. Subsistence farming, animal husbandry and fishing are the main economic activities in the area. Malaria is holoendemic in this area, and transmission occurs throughout the year. The ‘long rainy season’ from late March to May produces intense transmission from April to August. The ‘short rainy season’ from October to December produces another, somewhat less intense, transmission season from November to January.

### Study site

**Fig. 1:**
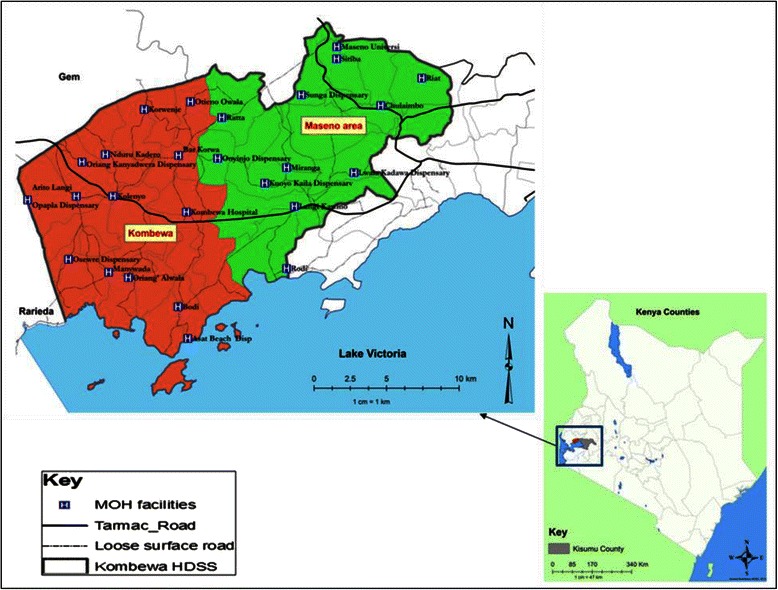
Location of the study site

### Study participants

The study sample was selected from Maseno Area within Kisumu County, western Kenya. Maseno Area is sub-divided into 4 locations, 17 sub-locations and 184 enumeration areas (villages) based on mapping work done earlier by the Kombewa Health and Demographic Surveillance System (Kombewa HDSS) run by the KEMRI/Walter Reed Project (34). The Kombewa HDSS is a longitudinal population registration system set up to monitor the evolving health and demographic problems of the study population in Kombewa and Maseno areas [34]. Some villages with less than 50 households were merged together to create new enumeration areas. A random sample of 7 households was drawn from each enumeration area, to give a projected sample of 1190 households, which with an estimated response rate of 85 % would give a total sample size of 1010. Village maps were used to assign households and guide the research assistants during the survey. Using the Kish Grid Method [35], one individual was selected from each of the sampled household. In the Kish Grid method, the researcher identifies all the adults in the household, places them into order of date of birth, and then selects one adult at random for interview using a grid of random numbers, tailored for household size. The demographics and reasons for the refusal were recorded in notebooks by the Research assistants. A total of 1190 households were visited.

### Study procedures

Meetings were held with community leaders to explain the purpose of the survey, and answer questions. The heads of the sampled households, and then the identified participants in the survey were approached in their own homes for informed written and witnessed consent to the interview. The interview was administered by one of a group of 20 research assistants using a personal digital assistant (PDA), on which the interview questions were programmed in English, Kiswahili and Dholuo, and interview responses were recorded. The research assistants received a 5 day training course. During the training a common understanding was developed and adopted of some of the technical words and phrases not commonly used locally. For example, there is no specific direct translation of the term “depression “in Dholuo and so depression was described using words such as “kuyo” “midhiero” and “parruok”. These words have a variety of meanings depending on the context and can refer to bereavement, dilemma and worry or thinking a lot respectively. The word “worry” was translated into the commonly used word in Dholuo “paro” which also means thinking. Thus we had to give examples of excessive worry. One of the Dholuo words used to describe irritability included “ger” which could also mean hostility or provocative.

Despite the difficulties in finding exact translations, the additional questions specified by the Clinical Interview Schedule Revised (see below) under each screening question were considered adequate to clarify the meaning of all such words. The research assistants were supervised daily in the field by a field manager.

The participants received a structured epidemiological assessment of common mental disorders, psychotic symptoms, alcohol and substance abuse, accompanied by additional sections on socio-demographic data, life events, social networks, social supports, disability/activities of daily living, quality of life, use of health services, and service use, adapted from the adult psychiatric morbidity schedule [36] used in the UK mental health survey programme.

Demographic information collected included age, sex, ethnicity, marital status and household status (head, spouse or other). Socio-economic factors assessed included employment status, education attainment, economic assets and type of housing.

Common mental disorders were assessed by the Clinical Interview Schedule- Revised (CIS-R) [37], a gold standard instrument for use by lay interviewers to assess psychopathology in community settings. It has been widely used in high [38–40] and low income countries [41–44], including Tanzania [45, 46] and Kenya [18]. The CIS-R measures the presence of 14 symptom-types in the preceding month and the frequency, duration and severity of each symptom in the past week. Scores, taken together with algorithms based on the ICD-10 [47], provide diagnoses of depressive episode (mild, moderate or severe), obsessive compulsive disorder, panic disorder, phobic disorder, generalised anxiety disorder and mixed anxiety/depressive disorder. In the previous studies in Tanzania [45, 46] and Kenya [18], and in this study, a score of 12 or more across the 14 sections of the survey was considered an indication of “any CMD”, as used in other CIS-R studies [38–40].

Respondents were given a list of 11 different stressful life events and asked to say which, if any, they had experienced in the last six months. The list included health risks (serious illness, injury or assault to self or close relative), loss of a loved one (death of a relative; death of a close friend), relationship difficulties (separation or divorce; serious problem with a close friend or relative); income instability (being made redundant or sacked; having looked for work for over a month; loss of the equivalent of three months income) and legal problems (problems with the police involving a court experience; something of value lost or stolen). The list was developed from the British psychiatric morbidity survey programme [38–40], and slightly modified for the east African context [18, 46]. Scores were grouped into “none”, “one”, “two” and “three or more” life events.

Perceived lack of social support was assessed from respondents’ answers to seven questions which were used in the 1992 Health Survey for England [48], and the British Surveys of Psychiatric Morbidity [38–40]. The seven questions take the form of statements that individuals could say were not true, partly true or certainly true for them in response to the question ‘There are people I know who’: (i) Do things to make me happy; (ii) Who make me feel loved; (iii) Who can be relied on no matter what happens; (iv) Who would see that I am taken care of if I needed to be; (v) Who accept me just as I am; (vi) Who make me feel an important part of their lives; and (vii) Who give me support and encouragement. Results were categorised into no, moderate or severe lack of perceived social support.

Social network size was assessed by respondents answers to three questions which have also been used in the ONS surveys of Psychiatric morbidity, namely (i) How many adults who live with you do you feel close to, (ii) how many relatives aged 16 or over who do not live with you do you feel close to, (iii) how many friends or acquaintances who do not live with you would you describe as close or good friends. Responses were added into a total social network score.

Specific questions were also asked about voluntary caring responsibilities to family or neighbours (Do you give care due to long term physical or mental disorder or disability? And if yes, Time spent giving care in a week). Questions were also asked about growing up with one natural parent or two until age 16; and about spending time in an institution such as a boarding school or orphanage before the age of 16.

### Statistical analysis

We examined the prevalence of CMD, individual diagnostic categories, individual non-psychotic symptoms, and of alcohol consumption, hazardous drinking and substance abuse. We also examined the predictors of CMD, using STATA version 11.2 [49] to calculate unadjusted and adjusted odds ratios. Households have been categorized into different socio-economic levels using an index of household assets, constructed applying the principal component analysis procedure, as a proxy indicator for socio-economic status. In developing the asset quintiles, type of house, roofing & walling material, source of water, toilet facility and land have been used [50, 51]. These individual items were not considered to be potential risk factors in their own right but rather simply as components of a poverty measure in a low income country. This approach is widely used in very poor settings where assets are considered to be a better appraisal of poverty than cash income which does not relate closely to assets [50, 51],

### Ethics

Ethical approval was granted by the Kings College London and Kenya Medical Research Institute Boards of Research Ethics respectively (PNM/11/12-54, SSC2374), and permission was obtained to conduct the study in households in Maseno area, which is part of the KEMRI/WRP Kombewa HDSS. Informed written and witnessed consent was asked of heads of sampled households, and then of sampled participants to take part in the study.

## Results

One thousand one hundred ninety households were selected, and 1158 participants consented to the study while 32 refused to participate in the study interviews, giving a response rate of 97.3 %.

Table 1 shows the prevalence of CMD in the last week, and the individual diagnostic breakdown.

**Table 1:** Prevalence of common mental disorders (CMDs)^a^ in the last week, using the combined category CMD (CIS-R score 12+) and the individual diagnostic groups which comprise CMD

	n	Prevalence %	95 % C.I
Total sample	1157		
Any CMD^a^	119	10.3	8.6 to 12.2
Specific CMD			
Mixed anxiety & depression	19	1.7	1.00 to 2.57
Panic disorder	35	3.1	2.13 to 4.21
Generalized anxiety disorder	8	0.7	0.30 to 1.37
Depressive episode	10	0.9	0.42 to 1.60
Phobic disorder	5	0.4	0.14 to 1.02
Obsessive compulsive disorder	16	1.4	0.80 to 2.25

*C.I* confidence interval

^a^Any CMD and specific CMDs in the past seven days as measured by the Clinical Interview Schedule-Revised (CIS-R); defined as anyone with a total symptom score of 12 or above

The prevalence of any CMD in the adult population was 10.3 (95 % confidence interval 8.6–12.2).

Table 2 shows the prevalence of any CMD by a range of socio-demographic, physical and psychosocial variables.

**Table 2:** Prevalence for any CMD over the last one week and its relationship with socio-demographic, psychosocial and health related factors using univariate analysis (odds ratios)

Factors		N	Prevalence (%)	Unadjusted OR (95 % C.I)	*p*-value
Any CMD		1157	156 (10.3)	8.59 to 12.18	
Sex	Male	604	23 (3.8)	1	–
	Female	548	96 (17.5)	5.4 (3.3 to 8.6)	<0.001
Age group	<30 years	282	21 (7.5)	1	–
	30–60 years	452	42 (9.3)	1.3 (0.7 to 2.2)	0.386
	>60 years	172	35 (20.4)	3.2 (1.8 to 5.7)	<0.001
Household size	<=6 people	568	50 (8.8)	1	–
	>6 people	584	69 (11.8)	1.4 (0.95 to 2.04)	0.094
Marital Status	Married/cohabiting	719	45 (6.3)	1	–
	Single	183	11 (6.0)	1.0 (0.5 to 1.9)	0.901
	Widowed/divorced	249	63 (25.3)	5.1 (3.3 to 7.7)	<0.001
Education	None	132	33 (25.0)	1	–
	Primary	627	67 (10.7)	0.4 (0.2 to 0.6)	<0.001
	Secondary	322	15 (4.7)	0.1 (0.08 to 0.28)	<0.001
	Post secondary	71	4 (5.6)	0.2 (0.06 to 0.53)	0.002
Employment status	Unemployed	566	51 (9.0)	1	–
	Self employed	487	61 (12.5)	1.4 (0.97 to 2.14)	0.066
	Employed	99	7 (7.1)	0.8 (0.34 to 0.53)	0.529
Asset Groups	Highest, Q1	402	26 (6.6)	1	–
	Q2	405	43 (10.7)	1.7 (1.02 to 2.83)	0.041
	Lowest, Q3	350	50 (13.8)	2.3 (1.38 to 3.73)	0.001
Perceived Social Support	No lack: 0	3	1 (33.3)	1	–
	Moderate lack: 1–7	313	47 (15.0)	0.4 (0.03 to 3.98)	0.400
	Severe lack: 8+	828	71 (8.6)	0.2 (0.02 to 2.09)	0.174
Total Life Event Score	0–3	833	73 (8.7)	1	–
	4 or more	309	46 (14.9)	1.8 (1.24 to 2.72)	0.003
Total Social Group Size	3 or less	144	19 (13.2)	1	–
	4 to 8	519	55 (10.6)	0.8 (0.45 to 1.36)	0.382
	9 or more	481	45 (9.4)	0.7 (0.38 to 1.20)	0.185
Being a carer for more than 4 h a week	No	26	2 (7.8)	1	–
	Yes	172	25 (14.5)	2.0 (0.45 to 9.18)	0.352
Spent time in an institution before age 16	No	920	88 (9.6)	1	–
	Yes	220	30 (13.6)	1.5 (0.96 to 2.33)	0.076
Did not have both natural parents at home until age 16	No	964	99 (10.3)	1	–
	Yes	176	19 (10.8)	1.1 (0.63 to 1.78)	0.833

*OR* odds ratio, *C.I* confidence interval

The risk of having any CMD was significantly higher in females compared to males (odds ratio = 5.4, *p* <0.001); in people aged 60 years and above compared to those below 30 years (odds ratio = 3.2, *p* < 0.001); in those who were widowed or divorced (odds ratio = 5.1, *p* < 0.001), those living in the lowest asset households (odds ratio 2.3 *p* = 0.001), and in those with 4 or more life events in the last six months (odds ratio 1.8, *p* = 0.003). Risk was reduced with increasing levels of education (odds ratio 0.4, *p* < 0.001 for primary education, OR 0.1, *p* < 0.001 for secondary education and OR 0.2, *p* = 0.002 for post secondary education and in those living in very large households, OR 0.7, p = 0.051). Having spent time in an institution before the age of 16 was of borderline significance (odds ratio 1.7, *p* = 0.076). Being a carer for more than 4 h a week had no significant impact on prevalence of CMD.

Those variables which were found to be significant in the unadjusted analyses were fed into an adjusted analysis (see Table 3).

**Table 3:** Relationship of any CMD with socio-demographic factors and health related factors, using logistic regression analysis, with odds ratios adjusted for presence of other factors significant at the bivariate level

Factors		Adjusted OR^a^ (95 % C.I)	*p*-value
Sex	Female	6.2 (3.25 to 11.71)	<0.001
Household size	>6 people	1.0 (0.61 to 1.75)	0.906
Marital status	Single	1.2 (0.47 to 3.08)	0.693
	Divorced/widowed	2.5 (1.37 to 4.42)	0.003
Age	30–60 years	1.1 (0.57 to 2.09)	0.797
	>60 years	2.3 (1.00 to 5.144)	0.052
Level of education	Primary	0.6 (0.33 to 1.23)	0.180
	Secondary	0.4 (0.17 to 1.12)	0.084
	Post secondary	0.5 (0.09 to 2.35)	0.347
Employment status	Self employed	3.2 (1.84 to 5.55)	<0.001
	Employed	3.3 (1.01 to 10.97)	0.049
Asset Groups	Q2	1.8 (0.97 to 3.42)	0.062
	Lowest, Q3	2.5 (1.35 to 4.82)	0.004
Total events life score	4 or more	1.0 (0.53 to 1.88)	0.991

*OR* odds ratio, *C.I* confidence interval

^a^variables identified as Univariate predictors of any CMD

From the adjusted analyses (see Table 3) there were significantly higher rates of having any CMD if one was female (OR 6.2, *p* < 0.001), divorced/widowed (OR 2.5, *p* < 0.003), aged over 60 (OR 2.3, *p* = 0.052), self employed (OR 3.2 *p* < 0.001), employed (OR 3.3, *p* = 0.049), or belonged to the lowest asset quintile (OR 2.5, *p* = .0.004). after adjusting for other variables significant at the bivariate level.

Table 4 shows the effect of age on CMD prevalence for each gender. It can be seen that the effect of age on CMD prevalence is significant for women (OR 2.3, *p* = 0.012 for age 30–60 years and OR 6.0, *p* < 0.001 for age >60) but not for men (OR 0.3, *p* = 0.052 for age 30–60 and OR 0.8, *p* = 0.787 for age >60).

**Table 4:** Effect of age on cmd prevalence in men and women

Age group	Males		Females	
	OR (95%C.I.)	*P* value	OR (95 %)	*P* value
<30 years	1		1	
30–60 years	0.3 (0.08–1.01)	0.052	2.3 (1.20–4.47)	0.012
>60	0.8 (0.24–2.96)	0.787	6.0 (2.94–12.23)	<0.001

Table 5 shows the change in CMD prevalence in each gender found between 2004 and 2013, using the same CIS-R interview for assessment of CMD.

**Table 5:** Changes in CMD prevalence between 2004 and 2013

		Prevalence: 2004	Prevalence: 2013	*p*-value*
Any CMD	Overall	10.8	10.3	0.716
	Male	10.9	3.8	<0.001
	Female	10.8	17.5	0.001

*p-value from test on equality of proportions

It can be seen that while the total CMD prevalence remained unchanged over the decade 2004–13, the prevalence for men dropped from 10.9 % to 3.8 % and the prevalence for women increased from 10.8 % to 17.5 %. Both changes were highly significant.

## Discussion

### Overall findings

The study found that the overall prevalence of CMD in 2013 was unchanged at 10.3 %. However there were significantly higher rates of having any CMD if one was female (OR 6.2, *p* < 0.001), divorced/widowed (OR 2.5, *p* < 0.003), aged over 60 (OR 2.3, *p* = 0.052), self employed (OR 3.2 *p* < 0.001), employed (OR 3.3, *p* = 0.049) or belonged to the lowest asset quintile (OR 2.5, *p* = .0.004) after adjusting for other variables significant at the bivariate level. The overall prevalence has been consistent over a decade time span, despite political and community turbulence. However, this apparent consistency masks the development of a striking difference in prevalence between the genders. Over the decade 2004-13, the prevalence for men dropped from 10.9 % to 3.8 % (*P* < 0.001) and the prevalence for women increased from 10.8 % to 17.5 % (*p* = 0.001).

### Comparison with other relevant studies

#### Prevalence and changes since 2004

Despite the serious election violence in Nyanza in 2007/8, the current study found a point prevalence of 10.3 % in 2013, largely unchanged from the prevalence of 10.8 % found in 2004 [18]. The total CMD in 2013 was comprised of mixed anxiety depression (6.1 %), panic disorder (2.6 %), generalised anxiety disorder (1.6 %) and depressive episodes (0.7 %), again unchanged from the diagnostic distribution in 2004. However, in 2004 we did not find a gender difference while in 2013 the higher prevalence in women compared to men is striking. Thus between 2004 and 2013, the prevalence in men has fallen from 10.9 % to 3.8 %, the prevalence in women has increased from 10.8 % to 17.5 %. We have not been able to find any other African studies reporting change over 10 years.

A study in the Yoruba-speaking part of Nigeria [52] was recently reported where the 12 month prevalence of any psychiatric disorder was 4.7 %, one of the lowest in 14 countries participating in the World Mental Health Surveys [53]. A survey in South Africa [54] found that the lifetime prevalence of DSM-IV/CIDI disorders was anxiety disorders (15.8 %), mood disorders (9.8 %), substance use disorders (13.4 %), and any disorder (30.3 %).

#### Risk factors for CMD

##### Gender

We found a highly increased prevalence in women compared to men. Other studies (Britain [55], Brazil [56], Zimbabwe [57], Ethiopia [58], and South Africa [59] have also found higher rates in women, but the previous study in Maseno [18] and the Nigerian study [52] failed to find a difference between genders. A pooled analysis of different countries participating in the world mental health survey has found higher rates of lifetime mood and anxiety disorders in women compared to men, although the gender ratios diminish with more recent country surveys [60].

It has been suggested that it is culturally more acceptable for women in any society to express their difficulties while men are expected to bear their problems with greater self control and to be reluctant to admit symptoms of distress. However it has been demonstrated in other studies that the higher rates of symptoms often reported by women reflect actual differences in symptom frequency and not the greater willingness of women to discuss their problems with others [61–63]. Indeed, controlling for three forms of response bias (naysaying, perceived trait desirability and the need for social approval actually increased the difference between the sexes [63]. Furthermore there is no reason to suppose that women would be relatively more likely than men to yea say in 2013 than in 2004.

In 2013, the survey interviewers were roughly equal numbers of men and women, whereas in 2004, the interviewers were mostly women, and so the gender ratio of interviewers differed between the two surveys. However, one would have expected that if the gender of the interviewer had an impact, then women would be more likely to disclose emotional issues to female than male interviewers, and so this change in the gender ratio of interviewers would have been expected if anything to reduce the relative rate of CMD detected in 2013 relative to that detected in 2004.

In this study, the gender ratio is surprisingly high and deserves further investigation. It may be that the high rates in women in this study are linked to the high prevalence of HIV in women in Nyanza Province [10], and its associated stress, or to other stressful issues such as experiencing domestic violence or violence outside the home. The experience of domestic violence in Nyanza is roughly 5 times more among women compared to men (49.5 % compared with 10.3 %). Moreover those women with low levels of education and greater levels of poverty were more at risk of domestic violence [64]. Generally women do not own property in the community studied so the perception of deprivation and less power to determine their destiny may make them more vulnerable to emotional distress. The 2013 Kenya population situation analysis reports an increase in absolute numbers of those living in poverty despite reports of improvement in overall economic performance in Kenya [65].

Women were found to have experienced higher rates of violence during the 2007–8 election related violence in Kenya [15]. However, the striking difference in prevalence between the genders may not be explained by the 2007–2008 post- election violence. The violence affected both males and females (though not to the same extent). If violence were the key factor causing the increase in women, it would then be expected that the prevalence CMD of the men would have gone up to some extent as well rather than be reduced. This study was carried out around the time of the 2013 election when there was no further election violence. We were surprised not to find an association with being a carer, as this is a major risk factor in high income countries [66]. Female Genital Mutilation is not practised in the Luo and Luhya tribes who inhabit the area of the survey.

A recent review of cross national associations between gender and mental illness pointed out that while epidemiological surveys have generally found higher rates of anxiety and mood disorder among women, evidence of decreasing gender differences have been found in countries where the roles of women have improved in terms of employment, access to birth control and other indicators of increasing gender role equality [60]. The striking increase in gender difference found in this study suggests there may be increasing rather than decreasing gender role inequality in this area of Kenya. It may be that the relevant inequality may be in the sheer load of responsibilities expected of each gender. Women residing in Siaya, Nyanza (especially those who are divorced, separated or widowed) are now taking the roles of sand harvesting and fishing, occupations only pursued by men previously [67]. Such roles are likely to be in addition to the usual domestic and child care responsibilities.

Even though Kenya ratified the UN Convention on the Elimination of All Forms of Discrimination against Women and has enacted legislation to implement its provisions in 1984, gender disparities remain widespread. Much of the problem lies with traditional practices that favour men in access to education, land, and inheritance, financial services, employment, and access to positions of political power [68]. From national data, there has been a significant rise in income since 2004, and it may be that this is disproportionately available to men rather than women. Indeed only 29 % of those who are formally employed are women [69]. It is worth noting that we have also found a similar gender ratio in prevalence of psychotic symptoms in this sample in 2013, again with similar changes between 2004 and 2013 [70].

##### Age

We found an increasing prevalence with age, with those aged over 60 having significantly increased rates compared to those under 30. This association with increased age was similar to the earlier findings in Kenya [18], South Africa [54] and Britain [71]. The effect of age on prevalence of CMD was largely confined to women, and it is possible that the rapid population growth is especially stressful for older women. In Nyanza, there are increasing numbers of older caregivers (usually women) to orphans whose parents died of HIV [72]. However, as indicated above, in this study we did not find an association of CMD with caregiving.

##### Marital status

There was no difference in prevalence between those who were married or single. This is similar to earlier findings in Kenya [18], but unlike in Britain [73] and in the World Mental Health Survey [60] which found that being stably married was associated with less risk compared to being single. We found that the prevalence was significantly increased for the widowed or divorced, similar to that in the world mental health survey overall [60], and in a number of developing countries [74] but unlike in the previous Kenya survey [18], or in Nigeria [52] and South Africa [54].

##### Education

The risk of any CMD was significantly reduced with increased levels of education, similar to surveys in Zimbabwe [57], Pakistan [75], Brazil [56], Burundi [76], and Britain [77].

##### Employment

In this survey we found that both the the employed and the self-employed had significantly higher rates of illness than the unemployed. This intruiging finding may be because in this poor area of Kenya, both employment and self employment usually imply being a rural farmer or fisherman subject to uncontrollable environmental factors while unemployment often implies a young person who has recently completed education and is still being cared for by parents in the family home.

In neighbouring Tanzania [46], as in Britain [78], the unemployed had significantly higher rates of disorder although the association did not remain after adjustment for other factors. Similarly in Zimbabwe, the association with unemployment was not consistent after adjustment for age and sex [57].

##### Assets

As income and expenditure are difficult to assess in poor rural subsistence farming communities, where income is seasonal, episodic, and produce may be eaten directly or exchanged rather than sold, we have followed the approach sometimes used elsewhere in development studies of appraising household assets to assess socio-economic level [50, 51].

Prevalence of CMD decreased with increasing assets, members of households in the highest asset quintile had a significantly lower risk of having any CMD when compared to those in the lowest asset quintile (odds ratio = 0.4). Others have also found a relationship of CMD with poverty [74].

##### Life events

The odds of experiencing CMD was significantly higher in those with 4 or more life events in the last six months at the bivariate level, similar to studies in Ethiopia [58], Zimbabwe [57] in SSA [60], and also in Pakistan [75], Brazil [56] and Britain [79], but this relationship disappeared in the adjusted analysis.

##### Social networks

We found no independent association of social network size with CMD, similar to previous findings in Tanzania [46] and South Africa [80]. In contrast, generally in Britain, those with low social network size are significantly more likely to have CMD [81]. However, a survey of various ethnic minority groups living in Britain found that large social network size was associated with the presence of CMD in Bangladeshi and Indian respondents but that there was no association with CMD in the other ethnic minority groups [82]. It may be that in relatively poor communities, those with a large social network find that the support which may be derived from the group is outweighed by the heavy demands made by large extended families for financial and practical support, especially for family weddings, funerals, medical and education expenses, as well as the emotional demands that large extended families may make. It is also noteworthy in this context that we did not find a relationship between CMD and perceived lack of social support, similar to previous findings in Tanzania [46].

### Strengths of study

The strengths of the study are the use of a health and demographic surveillance site for the random sample of households, the high response rate, and the systematic approach to the clinical and socio-demographic assessments. The population in the surveillance site is regularly monitored by field staff who visit each household bi-annually to capture health and demographic information (Birth rates, Death rates, Causes of Death, Pregnancies, Immunization status, in-and out-migrations, etc). Various studies nested on the DSS platform take advantage of the sampling frame inherent in the HDSS, whether at individual, household/compound or regional levels. This familiarity with survey procedures is likely to have been influential in the achievement of a high response rate.

### Limitations of study

The implementation of the study was hampered by a number of logistical challenges which included the difficult terrain, posing problems for local transport for research staff, and continuing administrative difficulties, which led to delays in the implementation of the project. The interviewing period, initially planned to last 3 months, took place over a period of 6 months, and was temporarily halted for several weeks over the period of the 2013 election due to further fears of election unrest.

The CIS-R has been validated in a number of high, middle and low income countries [83–91] on a variety of populations and has been shown to be a unidimensional measure of common mental disorder [92]. Its psychometric properties vary between countries and also depend on the choice of comparison (eg SCAN or CIDI or a non standardised psychiatric assessment). It has not yet been validated in Kenya, but as for the previous 2004 survey, it was carefully scrutinised by local clinicians for content validity within the local cultural context. Similarly, the other components of the adult psychiatric morbidity survey schedule (life events, social network size) have not been separately assessed for local validity. However all the sections of the adult psychiatric morbidity interview and its component scales used in this study and their individual items have been reviewed by local clinicians in Kenya and Tanzania in 2004 [18, 46] and again in Kenya in 2013 for this study, and considered to have content validity. As always, the potential for measurement error when using screening instruments should be acknowledged, given self-reported experiences may be subject to recall or social desirability or cultural response bias [93].

It is possible that the CIS-R did not adequately capture some of the symptoms in the local population. For example there are no direct translations into the local Dholuo language of words such as depression or irritability. Additionally in a rural community where most people labour in the farms, the term fatigue could have been misunderstood. We considered these issues in the translations into the local language and used culturally appropriate examples. We did not assess local cultural or religious practices. Religious conviction has been shown to be protective [94], and in Nyanza, religious practice, especially Christianity, is widespread. Most of the local Christian denominations also engage in healing rituals which may offer the local people additional relief from minor emotional disorders [95].

Due to the proximity of the Lake, fish is a staple food and omega 3 fatty acids are now known to be protective of depression [96].

## Conclusions

This repeat epidemiological study of CMD found that the overall prevalence of CMD was largely unchanged at 10.3 % in 2013, compared with 10.8 % in 2004. However, in 2013 there were significantly higher rates of having any CMD if one was female (OR 6.2, p <0.001), aged over 60 (OR 2.3, *p* = 0.052), divorced/widowed (OR 2.5, *p* < 0.003), self employed (OR 3.2, *p* < 0.001), employed (OR 3.3,*p* = 0.049 or belonged to the lowest asset quintile (OR 2.5, *p* = .0.004) after adjusting for other variables significant at the bivariate level.

Thus common mental disorders pose a significant public health burden in Kenya, and their overall prevalence has been generally consistent over a decade time span, despite a degree of political and community turbulence, similar to the consistency that has been found by repeat surveys in Britain in 1992, 2000 and 2007 [73]. However, this apparent consistency masks the development of a striking difference in prevalence between the genders. Over the decade 2004-13, the prevalence for men dropped from 10.9 % to 3.8 % (*p*  = 0.001) and the prevalence for women increased from 10.8 % to 17.5 % (*p* = 0.001).

The magnitude of the gender imbalance in this survey is particularly striking, especially as it was not apparent in the 2004 survey, and deserves further investigation. It may be that social changes over the decade in Kenya have been especially challenging for women.

This is a local rather than a national survey, and there is a need for a nationally representative mental health epidemiological survey in Kenya. The locality in which this survey was carried out is poor, and characterised by political unrest, high unemployment and environmental problems of drought, and water hyacinth in the Lake hampering the fishing industry.

The prevalence rate and risk factors found are important to inform mental health promotion and prevention programmes, public education and professional training programmes in relevant sectors. Future research could beneficially include consideration of the severity and impairment of activities of daily living caused by CMD and would contribute to understanding of the impact of CMDs on lives of this population.

## Abbreviations

CMD: Common mental disorder
KCL: Kings College London
KEMRI: Kenya Medical Research Institute
HDSS: Health and demographic surveillance systems
OR: Odds ratio
PDA: Personal digital assistant
